# Intraoperative localization of small pulmonary nodules to assist surgical resection: A novel approach using a surgical navigation puncture robot system

**DOI:** 10.1111/1759-7714.13234

**Published:** 2019-11-26

**Authors:** Gang Zhou, Xiangqian Chen, Baolong Niu, Yadong Yan, Fan Shao, Yubo Fan, Yu Wang

**Affiliations:** ^1^ School of Biological Science and Medical Engineering Beihang University Beijing China; ^2^ Beijing Advanced Innovation Center for Biomedical Engineering Beihang University Beijing China; ^3^ Department of Radiotherapy First Medical Center of Chinese PLA General Hospital Beijing China

**Keywords:** Navigation puncture robot, small pulmonary nodules, video‐assisted thoracoscopic surgery

## Abstract

**Background:**

Localization and resection of nonvisible, nonpalpable pulmonary nodules during video‐assisted thoracoscopic surgery is challenging. In this study we developed a surgical navigation puncture robot system in order to locate small pulmonary nodules before thoracoscopic surgery.

**Methods:**

Four pigs were divided into group A and group B and underwent positioning puncture with the aid of the robotic system. The pigs in group A breathed freely during the experiment, whilst mechanical ventilation was used on the pigs in group B.

**Results:**

Using the robotic system to locate nodules achieved good results. For group A, a total of nine simulated nodules were created and successfully localized. The mean positioning accuracy was 9.6 ± 4.9 mm (range, 3.2–17.4 mm), and the time required for system positioning was 7.1 ± 1.0 minutes (range, 5.6–8.2 minutes). For group B, a total of 23 simulated nodules were created and successfully localized. The mean positioning accuracy was 2.9 ± 1.5 mm (range, 0.7–5.9 mm), and the time required for system positioning was 7.8 ± 1.1 minutes (range, 6.3–9.7 minutes).

**Conclusions:**

The new method using a surgical navigation puncture robot system to locate small pulmonary nodules is feasible and safe, and its positioning accuracy is sufficient to meet clinical requirements. In addition, results indicated that breathing had a great influence on the positioning accuracy, mainly in the longitudinal direction. Our surgical navigation puncture robot system has wide future applications for accurately locating small pulmonary nodules in a clinical setting.

**Key points:**

**Significant findings of the study**: A new method using a surgical navigation puncture robot system was developed to locate small pulmonary nodules before thoracoscopic surgery. The results indicated that this method can provide accurate localization and permit smaller and more precise resections.

**What this study adds**: A surgical navigation puncture robot system has wide future applications for accurately locating small pulmonary nodules in a clinical setting.

## Introduction

Lung cancer is a malignant tumor that seriously endangers human health. According to the statistics of the National Cancer Center of China in 2015, the morbidity of lung cancer ranks first in men and second in women, while the mortality of lung cancer ranks first in both men and women.^1^ Advanced stage lung cancer at the time of diagnosis is one of the contributing factors to decreased survival in patients with lung cancer.^2^ Developing methods for detecting and accurately diagnosing early‐stage lung cancer is of paramount importance. With the widespread use of low‐dose computed tomography (CT) screening, detection of early lung cancer has become possible, and small solid nodules or ground‐glass nodules can be examined by screening.^3^

For small nodules that are suspected of being lung cancer, surgical removal of the lesion is necessary. Only surgery can remove intact lesions and obtain sufficient tissue volume for accurate pathological diagnosis, pathological staging and molecular identification.^4^, ^5^ Since the advent of video‐assisted thoracoscopic surgery (VATS), it has been widely used for the diagnosis and treatment of small pulmonary nodules due to its safe and minimally invasive nature.^6^ However, a major problem with conducting successful thoracoscopic resection is locating small or deeply situated target nodules, which cannot be identified by inspection or palpation and often are difficult to visualize by thoracoscopy.^7^, ^8^, ^9^ Therefore, locating small pulmonary nodules in advance is a key step in the operation. Accurate localization of small pulmonary nodules will greatly improve the success rate of the operation.^10^, ^11^

Many studies have reported localization techniques of small pulmonary nodules before minimally invasive resection. Currently, the most widely used localization technique in clinical practice is to place positioning markers, such as hookwires,^12^, ^13^ microcoils,^14^, ^15^ methylmethine,^16^ and radiotracers,^17^, ^18^ under the guidance of CT before surgery and then remove the lesion according to the position of the marker during surgery. These localization techniques have been indicated to be very helpful in clinical practice. However, in most hospitals, the CT scanner is separate from the operating room, and the preoperative positioning process takes place in the CT room; however, minimally invasive surgery occurs in the operating room and patients may be at risk of marker migration, contamination, and lung injury during transportation from the CT room to the operating room.^2^, ^19^ The optimal solution is that nodule localization is also completed in the operating room without the guidance of CT, thus ensuring that localization and resection are in the same environment, and there is no requirement to move the patient.

This study proposes a new method for intraoperative localization of small pulmonary nodules. With our self‐developed surgical navigation puncture robot system, small pulmonary nodules can be accurately located in the operating room. Positioning markers are then placed near the nodules to assist thoracoscopic surgical resection. Animal experiments were carried out in our study to evaluate the feasibility, safety and accuracy of the new method.

## Methods

### Surgical navigation puncture robot system

**Figure 1:**
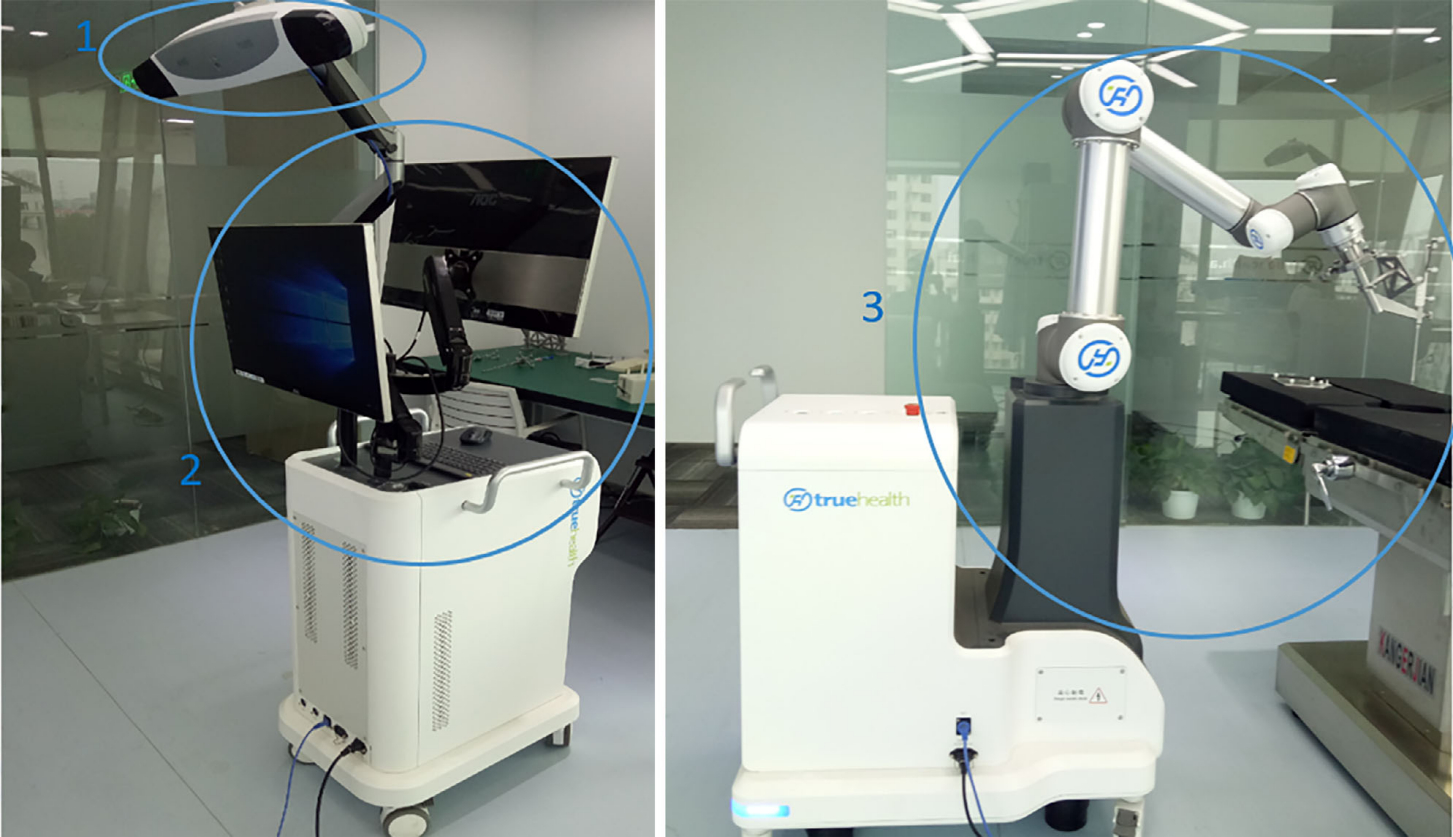
Surgical navigation puncture robot system (1) photoelectric navigation system, (2) software planning system, (3) robotic arm positioning and puncture system).

A surgical navigation puncture robot system, shown in Fig 1, was developed by Beijing TrueHealth Medical Technology Co., Ltd. The whole system is divided into three parts: (i) A photoelectric navigation system; (ii) A software planning system and (iii) A robotic arm positioning and puncture system.

**Figure 2:**
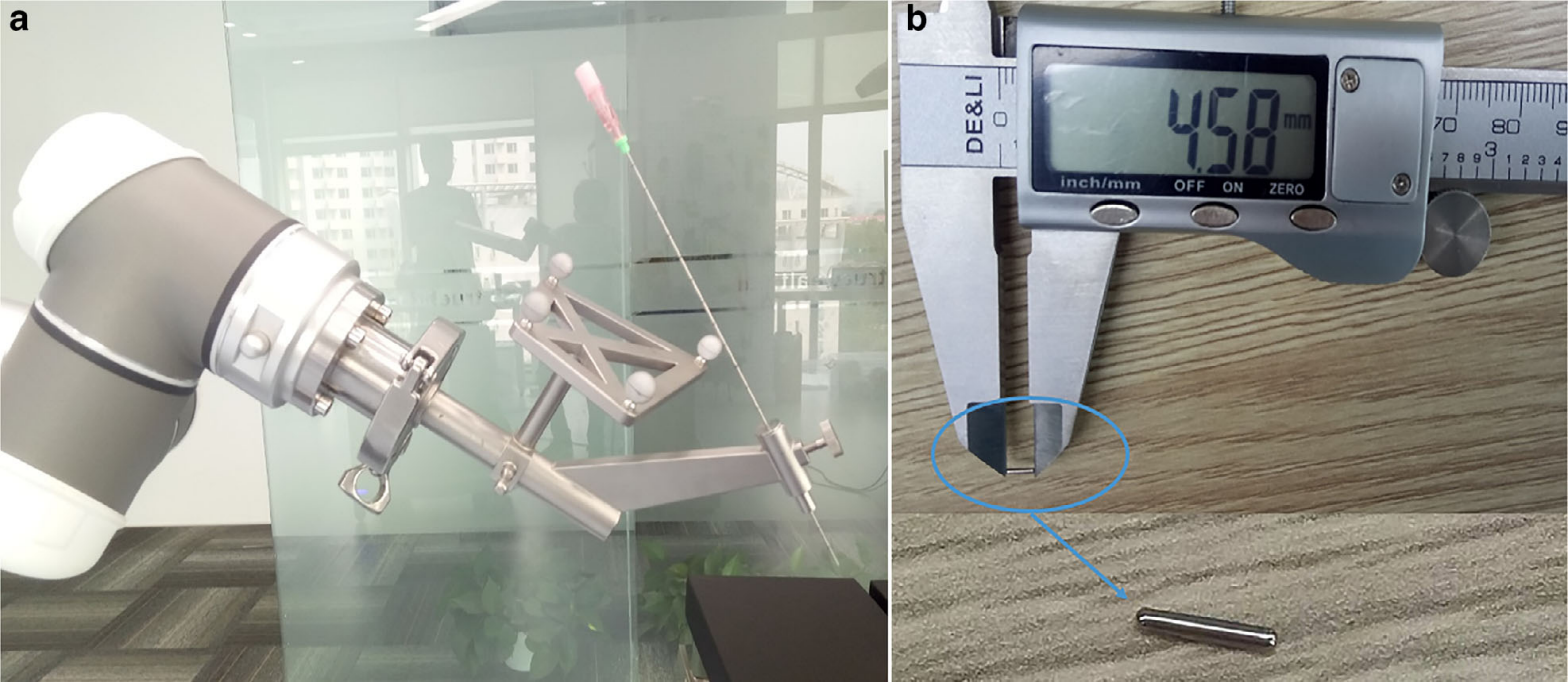
(**a**) The end sleeve of the arm and the puncture needle. (**b**) Titanium fiducial marker, 0.8 × 4 mm.

The operation principle of the surgical navigation puncture robot is as follows: (i) The patient's preoperative CT is obtained in advance to reconstruct a three‐dimensional (3D) model of pulmonary nodules, blood vessels, bronchi, ribs, and skin, which are imported into the software planning system; (ii) In the operating room, the position information of the patient obtained through the photoelectric navigation system is registered with the 3D model in real time; (iii) The physician completes the puncture path planning in the software planning system according to the position of the nodule in the 3D model; (iv) The robotic arm positioning system fixes the planned puncture path (including the needle insertion point, needle insertion angle and needle insertion depth) in the surgical space; (v) The physician inserts the puncture needle into the patient's small pulmonary nodule with the aid of the robotic arm to place the marker, which can be a hookwire, microcoil wire, medical glue, etc. The whole small pulmonary nodule positioning process is then completed. The end sleeve of the arm and the puncture needle are shown in Fig 2a:

### Animal care

Animal care, housing, and surgery were performed with the approval of the animal welfare ethics committee of Beijing Shichuang Century Miniature Pig Breeding Base. Four pigs (age: <1 year; bodyweight: 25.5–32.5 kg) were used in this study and were divided into groups A and B, of which group A was pig No. 1 and No. 2 and group B was pig No. 3 and No. 4. All pigs were premedicated by intramuscular injection of 0.04 mg/kg atropine sulfate before the experiment commenced. They were then sedated with an intramuscular injection of 20 mg/kg ketamine hydrochloride and 2 mg/kg xylazine hydrochloride. Animals were then intubated with a dual‐lumen endotracheal tube through an incision in the trachea.

### Experimental procedure

#### Simulated nodule formation

**Figure 3:**
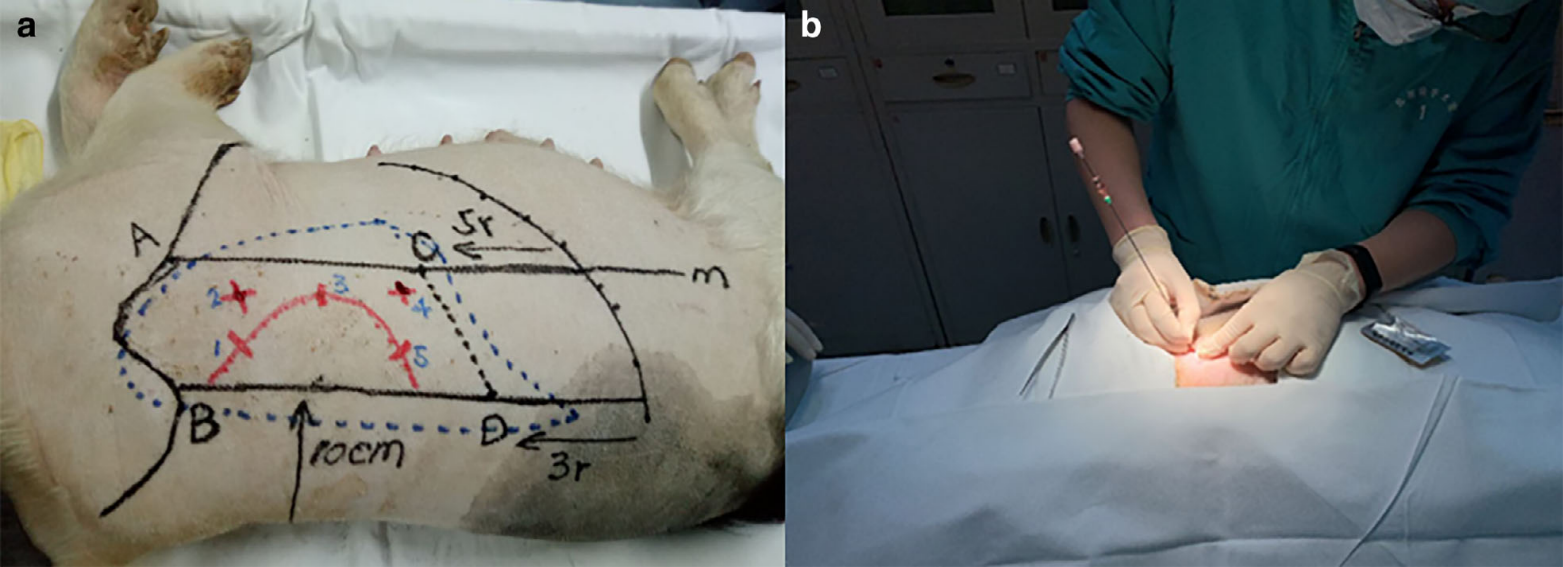
Simulated nodule placement.

In this experiment, a 0.8 × 4.5 mm titanium fiducial marker, depicted in Fig 2b, was used as a simulated nodule. The lungs of the pigs were scanned by CT to confirm that they were intact and an outline of their lungs was marked on their chest (Fig 3a). A titanium fiducial marker was placed percutaneously into the lung of the pig through an 18‐gauge needle, as shown in Fig 3b, so that a simulated nodule formed. In the two pigs in group A, five nodules were placed in the right lungs, and in the two pigs in group B, six nodules were each placed in the right lung and the left lung.

#### CT scan and 3D reconstruction

**Figure 4:**
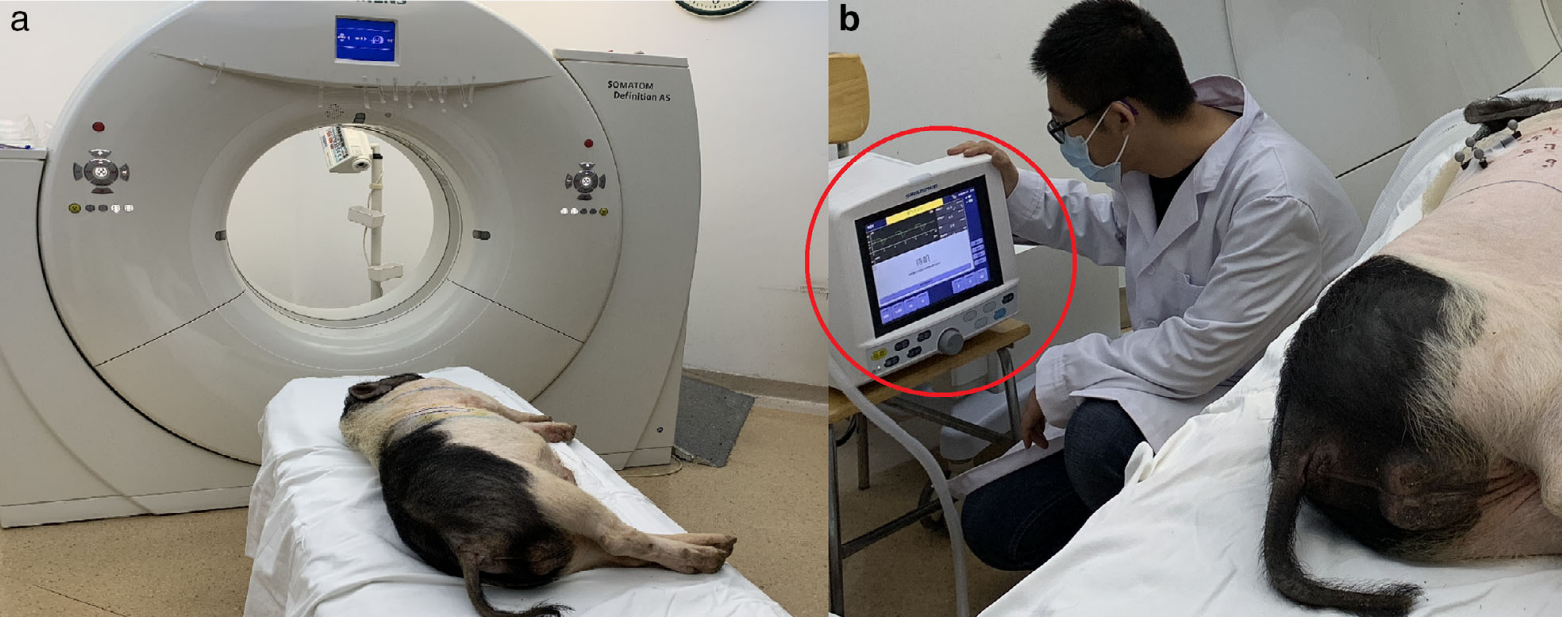
CT scan.

**Figure 5:**
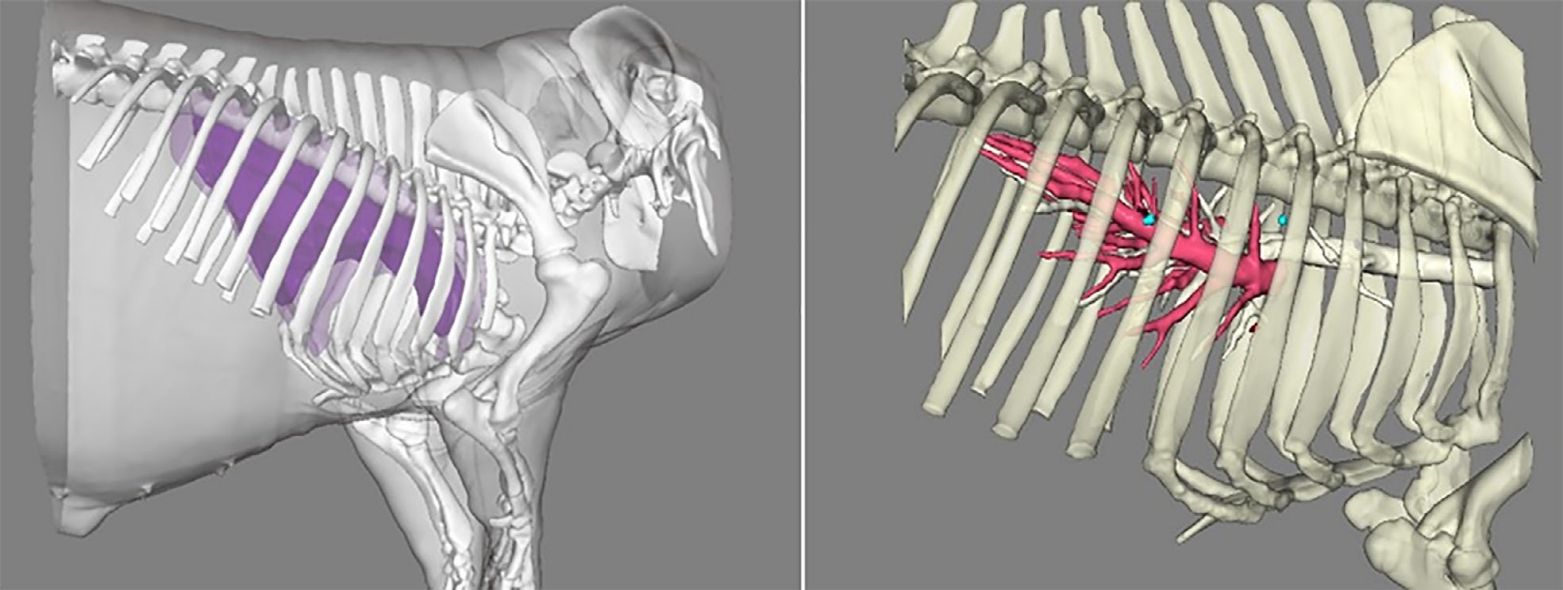
Three‐dimensional reconstruction.

After the simulated nodule was placed, the pig was placed in the lateral position and the metal positioning points (special alloy particles with a diameter of 1 mm without artifacts under CT) were attached to the surface of the pig's skin. A CT scan (Siemens SOMATOM Definition AS) was then performed to obtain the Dicom data of the lung. During the scan, pigs in group A breathed freely as shown in Fig 4a, and the inhalation of pigs in group B was controlled using an R30 ventilator (Beijing Siriusmed Medical Device Co., Ltd.) as shown in Fig 4b. The obtained CT Dicom data were reconstructed to obtain 3D models of the skin, ribs, lungs, simulated nodules, blood vessels and bronchi as shown in Fig 5.

The specific method of using a ventilator to control breathing was as follows: after the pig was intubated, the ventilator was attached and the pig's respiratory rate and inspiratory volume were monitored. After a while, the ventilator gradually replaced the pig's spontaneous breathing. After breathing was stable, the pig's breathing was completely controlled by the ventilator and there was no spontaneous breathing. At this time, the respiratory rate and maximum inspiratory volume of the pig could be observed through the ventilator. When a CT scan was required, the gas flow was cut off and the pig's breath controlled through the ventilator when the screen of the ventilator indicated a maximum inspiratory volume. The end of the inhalation represented the moment of maximum inspiratory volume.

#### Navigation planning and robot assisted positioning puncture

**Figure 6:**
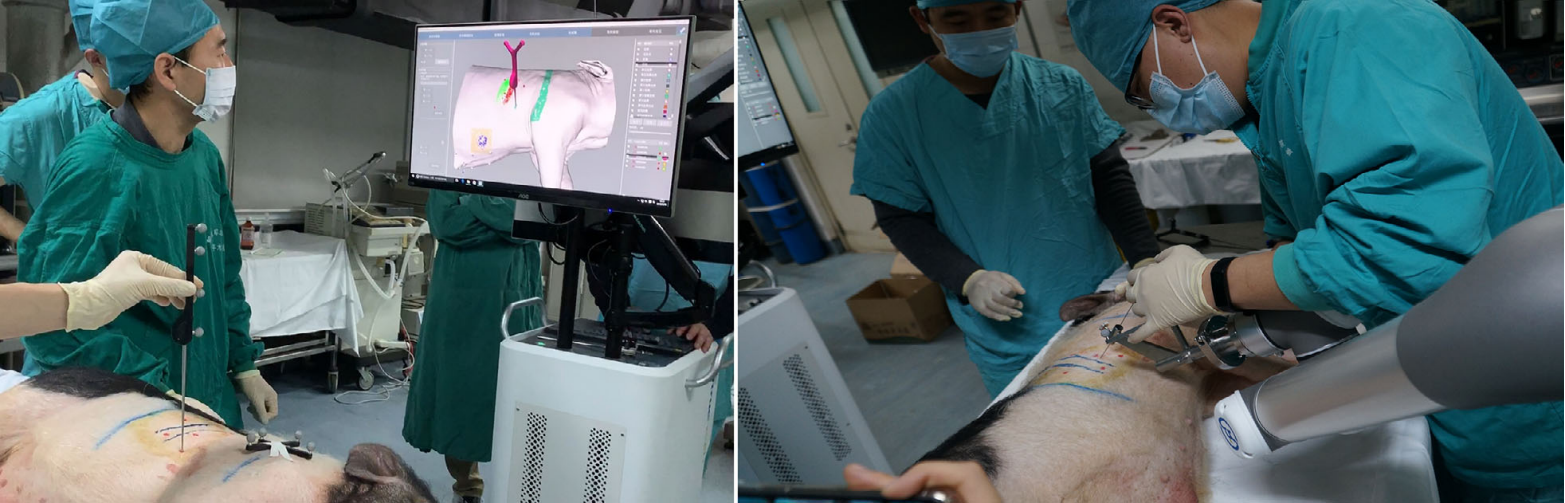
Navigation planning and robot assisted positioning puncture.

In the operating room, the pigs were placed in the same position as for the CT scan. The reconstructed 3D models were then imported into the software planning system, and the position of the pig in the surgery space was registered with 3D models by the body surface's metal positioning points. The puncture path was planned according to the virtual nodule position in the 3D model, and the robot arm accurately performed automatic positioning according to the planned path. After positioning was completed, the physician made a puncture using an 18‐gauge needle with the aid of the robotic arm and inserted the positioning marker, as shown in Fig 6. The positioning marker used was also a 0.8 × 4.5 mm titanium fiducial marker, as shown in Fig 2b. During the navigation and positioning puncture, the pigs in group A were in a state of free breathing, and the pigs in group B were controlled by ventilator, and the method of breath control by ventilator was consistent with the control under CT. For group B, during this period it was necessary to control breathing at the end of inhalation twice: once during registration when the robotic arm was performing automatic positioning, and secondly when the surgeon punctured the nodule with the aid of the robotic arm to insert the positioning marker.

#### CT scan to verify accuracy

After positioning had been completed, the pigs were again subjected to CT scan to obtain CT data of the lung. The spatial distance between the simulated nodule and the position marker was measured on the scan and the measured distance was the navigation positioning accuracy of the surgical navigation puncture robot system.

## Results

The pigs in the experiment were divided into group A and group B. The purpose of group A was to measure the positioning accuracy of the surgical navigation puncture robot system when the pigs were freely breathing, and the purpose of group B was to measure the positioning accuracy of the surgical navigation puncture robot system when the pigs underwent mechanical ventilation. No pneumothorax occurred during the localization of all nodules, and puncture paths planned by the physician with the aid of the robot system accurately avoided the blood vessels and no obvious bleeding symptoms occurred. The whole robot assisted positioning puncture process was safe and effective. The specific results are detailed below.

### Group A

A total of 10 simulated nodules were created in the two pigs in group A. Of the 10 simulated nodules, a simulated nodule of pig No. 2 was placed in the skin, not in the lung, when performing the CT scan. Excluding the invalid data, a total of nine nodules were successfully placed, and the positioning was completed. The average distance between the nine nodules and the visceral pleura was 14.1 ± 7.0 mm. The positioning errors are shown in Table 1. The mean of the positioning error was 9.6 mm (range: 3.2–17.4 mm), and the time required for system positioning ranged from 5.6 to 8.2 minutes (mean: 7.1 minutes) from the time the reconstructed 3D models were imported until the robot assisted puncture was completed.

**Table 1:** The positioning accuracy of Group A

		Nodule	x‐axis	y‐axis	z‐axis	Total error
Pig No. 1	Right lung	1	2.1	1.3	2.0	3.2
		2	2.3	3.5	0.0	4.2
		3	6.8	1.0	9.0	11.3
		4	4.7	1.0	12.8	13.7
		5	7.1	5.2	15.0	17.4
Pig No. 2	Right lung	1	3.0	0.8	2.0	3.7
		2	6.0	1.0	12.8	14.2
		3	1.0	6.5	9.0	11.1
		4	2.0	1.8	6.7	7.2
Mean			3.9	2.5	7.7	9.6
Standard deviation			2.2	2.0	5.1	4.9

The x‐axis was the direction from the back of the pig to the abdomen; the y‐axis was the direction from the right side of the pig to the left side; and the z‐axis was the direction from the tail of the pig to the head; unit, mm.

The positioning errors for group A in the three directions of x, y and z, respectively were 3.9 mm (1.0–7.1 mm), 2.5 mm (1.0–6.5 mm), and 7.7 mm (0.0–15.0 mm).

### Group B

**Figure 7:**
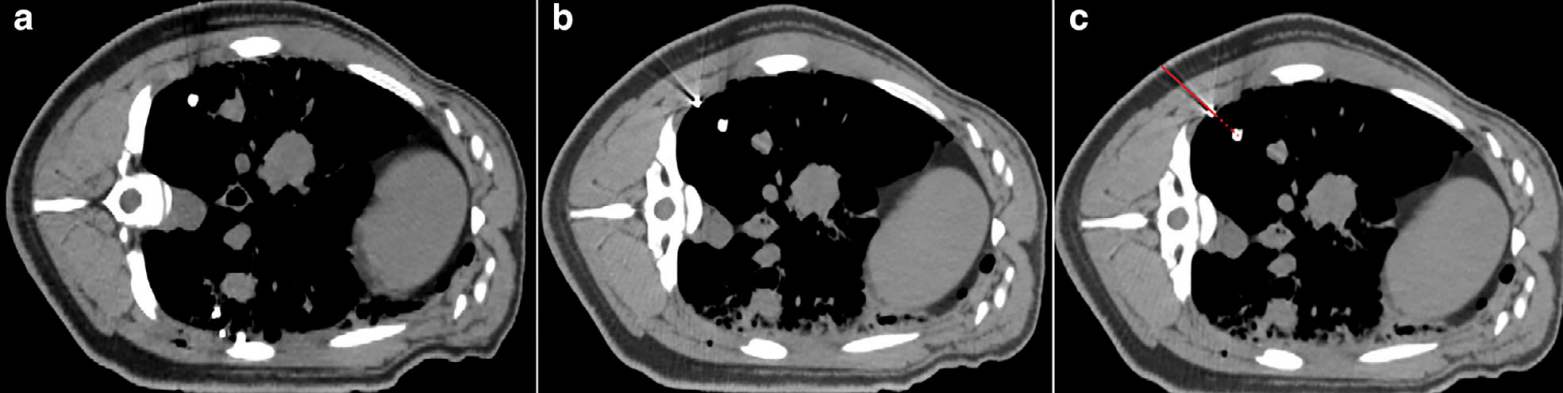
(**a**) The simulated nodule. (**b**) The simulated nodule and the positioning marker. (**c**) The needle‐path extension line.

A total of 24 simulated nodules were created in the two pigs in group B. Upon CT verification after positioning was completed, a positioning marker of pig No. 4 slid down the lung surface along the needle path, as shown in Fig 7b and the CT before positioning was showed in Fig 7a. Because the position of the needle‐path could be seen on the CT, further analysis showed that the needle‐path extended just down the needle track, and the extension line of the needle‐path could just pass through the simulated nodule, as shown in Fig 7c. This means the positioning was actually accurate, but the positioning marker slid down the needle track when the needle was removed.

Excluding the invalid data, a total of 23 nodules were successfully placed, and the positioning was completed. The average distance between the 23 nodules and the visceral pleura was 10.4 ± 5.0 mm. The positioning errors were as shown in Table 2. The mean of the positioning error was 2.9 mm (range: 0.7–5.9 mm), and the time required for system positioning ranged from 6.3 to 9.7 minutes (mean: 7.8 minutes) from the time the reconstructed 3D models were imported to completion of the robot assisted puncture.

**Table 2:** The positioning accuracy of Group B

		Nodule	x‐axis	y‐axis	z‐axis	Total error
Pig No. 3	Right lung	1	2.4	0.6	2.0	3.2
		2	1.9	1.8	2.6	3.7
		3	2.2	3.5	0.5	4.2
		4	1.0	0.6	3.0	3.2
		5	0.2	0.6	1.0	1.2
		6	0.1	0.5	0.9	1.0
	Left lung	1	3.4	2.7	4.0	5.9
		2	0.1	3.2	0.5	3.2
		3	0.9	0.8	2.0	2.3
		4	1.5	1.7	0.2	2.3
		5	1.8	1.2	0.5	2.2
		6	2.3	1.0	1.5	2.9
Pig No. 4	Right lung	1	0.5	0.5	2.0	2.1
		2	2.8	2.9	1.0	4.2
		3	0.6	0.4	0.0	0.7
		4	4.3	0.5	1.0	4.4
		5	0.8	0.3	0.0	0.9
	Left lung	1	1.2	1.4	0.6	1.9
		2	0.4	0.2	0.2	0.5
		3	1.8	0.5	1.0	2.1
		4	3.0	1.5	3.5	4.8
		5	1.4	0.9	5.0	5.3
		6	3.3	2.9	1.0	4.5
Mean			1.6	1.3	1.5	2.9
Standard deviation			1.2	1.0	1.3	1.5

The x‐axis was the direction from the back of the pig to the abdomen; the y‐axis was the direction from the right side of the pig to the left side; and the z‐axis was the direction from the tail of the pig to the head; unit, mm.

The positioning errors for group B in the three directions of x, y and z, respectively were 1.2 mm (0.1–4.3 mm), 1.0 mm (0.2–3.5 mm), and 1.3 mm (0.0–5.0 mm).

**Figure 8:**
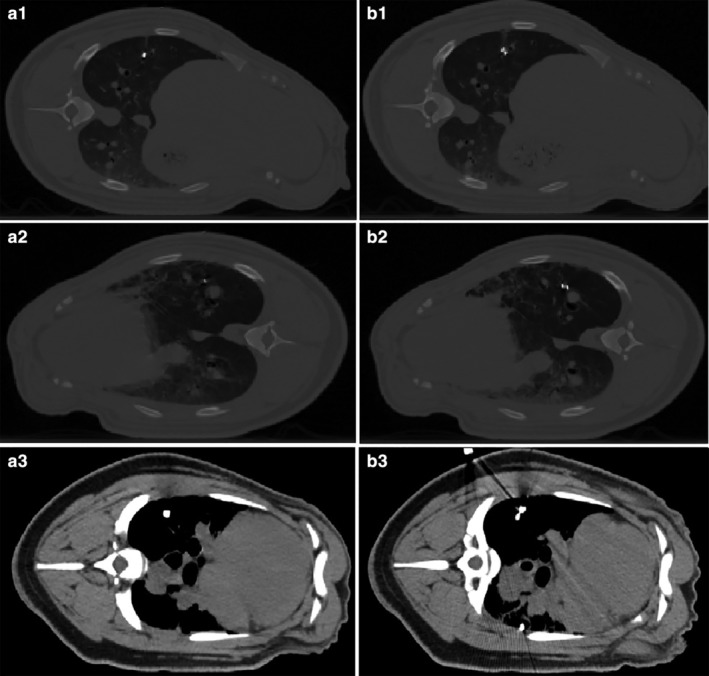
(**a1**) A simulated nodule for the right lung of pig No. 3 before the positioning puncture. (**b1**) The simulated nodule and positioning marker for the right lung of pig No. 3 after the positioning puncture. (**a2**) A simulated nodule for the left lung of pig No. 3 before the positioning puncture. (**b2**) The simulated nodule and positioning marker for the left lung of pig No. 3 after the positioning puncture. (**a3**) A simulated nodule for the right lung of pig No. 4 before the positioning puncture. (**b3**) The simulated nodule and positioning marker for the right lung of pig No. 4 after the positioning puncture.

Some CT results before and after positioning puncture are shown in Fig 8.

## Discussion

The results indicate that when the pigs underwent mechanical ventilation, the positioning accuracy of the whole system was 2.9 mm (range: 0.7–5.9 mm). Considering the actual clinical resection process, the cutting margin of the small pulmonary nodules was greater than 1 cm. This positioning accuracy was sufficient to meet the clinical positioning requirements. In addition, the entire positioning puncture process took 6.9 minutes (range: 4.1–9.8 minutes), which is much shorter than the time spent on a normal surgery of a thoracoscopic resection of small pulmonary nodules. According to reports in the literature,^20^, ^21^ the average operating time for thoracoscopic resection is approximately 50 minutes. Adding the time spent on anesthesia, disinfection and other preoperative preparations, a complete thoracoscopic resection surgery for a small pulmonary nodule takes on average approximately two hours. This means the time for positioning can be easily accepted in the clinical setting. For the 24 cases of simulated nodules, except for one case that was off target, all were successfully located and the success rate of positioning was 95.8%. Therefore, this animal experiment successfully verified the feasibility and safety of a new method by using a surgical navigation puncture robot system and achieved satisfactory results in terms of accuracy, which lays a solid foundation for further clinical application.

Moreover, the pigs in this experiment were divided into group A and group B to investigate the effect of breathing on the accuracy of the new positioning method. The experimental results showed that the positioning accuracy was 9.6 mm when mechanical ventilation was not performed, while the positioning accuracy increased to 2.9 mm after breathing was controlled. This finding indicates that the respiratory motion of the lung has great influence on the positioning accuracy, and it is a key factor in determining whether the positioning is accurate. In addition, when analyzing the uniaxial error of pig No. 1 and pig No. 2 in Group A, the x‐axis was 3.9 mm (1.0–7.1 mm); the y‐axis was 2.5 mm (1.0–6.5 mm); and the z‐axis was 7.7 mm (0.0–15.0 mm). Obviously, the largest direction of error was in the z‐axis, and the z‐axis corresponds to the head to tail direction of the pig. This observation shows that the most important effect of breathing on the lung is in the longitudinal direction, and the other two directions have less influence.

For group A, the positioning accuracy of nodules 1 and 2 of pig No. 1 and nodule 1 of pig No. 2 was much higher than the remaining nodules. The average positioning error of these three nodules was 3.7 mm, and the error in the three axial directions was relatively average. The reason for this was that these three nodules were located in the upper part of the pigs' lungs, which was less affected by breathing, thus resulting in a higher positioning accuracy. The 3.7 mm positioning accuracy of these three nodules was close to the average positioning accuracy of that in group B which was 2.9 mm. This finding shows that the effect of breathing on the upper lung positioning accuracy was not so significant. For group B, after breathing was controlled, the average positioning error of the 23 nodules was 2.9 mm, and the errors in the x‐axis, y‐axis, and z‐axis were relatively close (1.6 mm, 1.3 mm, and 1.5 mm, respectively) and they tended to have a uniformly distributed random error. This finding means that after breathing is controlled, the positioning error is relatively uniform and random, achieving a satisfactory result.

In summary, this study demonstrated the safety and feasibility of a new method, and the results of animal experiments show that this method can provide accurate localization and permit smaller and more precise resections. We believe the new method has great potential in clinical use. The procedure does not require CT fluoroscopic guidance, so there is no additional radiation exposure to patients and surgeons. In addition, the positioning and operation are completed in the operating room. The patient does not need to move, thus avoiding the risk of marker displacement and lung injury when the patient is transferred from the CT room to the operating room, and coordination between the CT room and the operating room is not required, thereby greatly improving efficiency. Moreover, most location techniques use preoperative CT‐guided percutaneous punctures with local anesthesia, so the patient is awake during the procedure. During the puncture, if the patient feels discomfort due to tension or pain, they will move which interfers with the puncture process. However, if the new method is introduced into clinical practice, it will be performed under general anesthesia. The puncture will go smoothly, and there will be no concern about whether the patient will be cooperative. Therefore, we firmly believe that our new method can be used perfectly for VATS in the treatment of small pulmonary nodules, and in the future we will undertake further clinical trials to confirm its reliability and usefulness.

## Disclosure

No authors report any conflict of interest.
